# Improving Medication Adherence Through Adaptive Digital Interventions (iMedA) in Patients With Hypertension: Protocol for an Interrupted Time Series Study

**DOI:** 10.2196/24494

**Published:** 2021-05-12

**Authors:** Kobra Etminani, Carina Göransson, Alexander Galozy, Margaretha Norell Pejner, Sławomir Nowaczyk

**Affiliations:** 1 Center for Applied Intelligent Systems Research Halmstad University Halmstad Sweden; 2 Center for Research on Welfare, Health and Sport Halmstad University Halmstad Sweden; 3 Hemvårdsförvaltningen Halmstad Sweden

**Keywords:** medication adherence, hypertension, digital intervention, mHealth, artificial intelligence

## Abstract

**Background:**

There is a strong need to improve medication adherence (MA) for individuals with hypertension in order to reduce long-term hospitalization costs. We believe this can be achieved through an artificial intelligence agent that helps the patient in understanding key individual adherence risk factors and designing an appropriate intervention plan. The incidence of hypertension in Sweden is estimated at approximately 27%. Although blood pressure control has increased in Sweden, barely half of the treated patients achieved adequate blood pressure levels. It is a major risk factor for coronary heart disease and stroke as well as heart failure. MA is a key factor for good clinical outcomes in persons with hypertension.

**Objective:**

The overall aim of this study is to design, develop, test, and evaluate an adaptive digital intervention called iMedA, delivered via a mobile app to improve MA, self-care management, and blood pressure control for persons with hypertension.

**Methods:**

The study design is an interrupted time series. We will collect data on a daily basis, 14 days before, during 6 months of delivering digital interventions through the mobile app, and 14 days after. The effect will be analyzed using segmented regression analysis. The participants will be recruited in Region Halland, Sweden. The design of the digital interventions follows the just-in-time adaptive intervention framework. The primary (distal) outcome is MA, and the secondary outcome is blood pressure. The design of the digital intervention is developed based on a needs assessment process including a systematic review, focus group interviews, and a pilot study, before conducting the longitudinal interrupted time series study.

**Results:**

The focus groups of persons with hypertension have been conducted to perform the needs assessment in a Swedish context. The design and development of digital interventions are in progress, and the interventions are planned to be ready in November 2020. Then, the 2-week pilot study for usability evaluation will start, and the interrupted time series study, which we plan to start in February 2021, will follow it.

**Conclusions:**

We hypothesize that iMedA will improve medication adherence and self-care management. This study could illustrate how self-care management tools can be an additional (digital) treatment support to a clinical one without increasing burden on health care staff.

**Trial Registration:**

ClinicalTrials.gov NCT04413500; https://clinicaltrials.gov/ct2/show/NCT04413500

**International Registered Report Identifier (IRRID):**

DERR1-10.2196/24494

## Introduction

### Overview

Hypertension is a common, dangerous, and treatable but undertreated condition worldwide and has a prevalence of approximately 30% in adults [1]. It becomes more common in more advanced ages, and the prevalence increases to 60% in persons over 60 years of age [2]. However, about 33% of persons with hypertension are unaware of their condition [2-4]. The incidence of hypertension in Sweden is estimated at approximately 27% [5]. Although blood pressure (BP) control has increased in Sweden, barely half of the treated patients achieved adequate BP levels [6].

Hypertension causes increased mortality and morbidity, most often by congestive heart failure, ischemic heart disease, and ischemic and hemorrhagic cerebrovascular insults [7]. Hypertension is also associated with increased occurrence of peripheral vascular disease and chronic kidney disease [8,9].

According to the National Board of Health and Welfare in Sweden [10], support and motivation for lifestyle changes as well as for self-care management are the first options when a person is diagnosed with hypertension, that is, having a systolic BP >140 mm Hg and diastolic BP >90 mm Hg measured repeatedly several times (3-6) over weeks or months, after 5 minutes of rest, while sitting with the right arm at heart level [10-12].

Lifestyle changes such as reducing weight, quitting smoking, reducing alcohol, and increasing physical activity are usually supported by visits to the district nurses individually or in groups in order to lower the BP. The support consists of educational and behavioral strategies to motivate and facilitate the person to conduct lifestyle changes in daily life [10]. If the lifestyle changes do not give results in lowering the person’s BP, medication treatment is required.

### Low Adherence to Medication

The World Health Organization defines adherence to long-term therapy as “the extent to which a person's behavior—taking medication, following a diet, and/or executing lifestyle changes—corresponds to agreed recommendations from a health care provider” [13]. In this work, we focus specifically on adherence to a medication regimen to improve BP control.

Although there are several effective medications that prevent cardiovascular events, persons can have poorly regulated BP. The main reasons for poor regulation of BP include patients’ lack of adherence to treatment and a lack of monitoring and intervention on the part of the doctor [2,14,15].

Adherence to the medication has been shown to be deficient. As an example, an analysis of pharmacy records has demonstrated that less than 50% of the study population had collected prescribed medications intended for hypertension [2]. Adherence to treatment is affected by many factors, including polypharmacy (ie, the number of tablets prescribed for hypertension), practical difficulties (eg, managing treatment costs, forgetfulness), and personal impressions affecting adherence (eg, side effects of medications) [16].

### Factors Affecting Adherence

Previous studies have linked both intrinsic and external factors to low or poor medication adherence (MA). Intrinsic factors include demographics such as age and income [17,18]. Psychological determinants include perceived susceptibility [19,20] and trust in the care provider [21,22], and behavioral factors include forgetfulness [19,23], employment, and travel [23]. External factors include the number of medications prescribed [18], availability of prescribed medication [23], and family support [18].

### Intervention Strategies That Improve Medication Adherence

A person's adherence can be improved by affecting the person's habit patterns in concurrence with the medication administration [24]. With BP monitoring at home, provision of adequate feedback, and improvement of cooperation between the person and health care personnel, the likelihood of the person retrieving their medication at the pharmacy increases [25].

There are several recommended approaches to address MA. Education strategies directed to the person have been shown to be of great importance [26]. Several studies have reported that health literacy levels may predict MA [27-29]. Behavioral strategies are important interventions to improve adherence to medications [26,30]. These strategies can vary and include unit-dose packaging, self-monitoring of medication, self-monitoring of symptoms or side effects, reminders, and other cues to action such as associating medication-taking with other daily activities.

Educational and behavioral strategies are highlighted in guidelines but are lacking in how to support the persons to improve MA [30]. In general, interventions successful in improving BP control and increasing MA are ones that improve awareness of and involvement in the treatment [31].

### Digital Interventions for Medication Adherence

Modern technologies make it possible to reach a vast number of persons using few personnel resources. With the widespread use in Sweden of the Internet and smartphones (93.1% internet penetration [32]), there are opportunities to use digital interventions, in specific to support people with hypertension on a wide scale and at low cost.

The authors have performed a systematic review recently on studies that applied digital interventions in order to improve BP and MA during the past decade [33]. The results show that digital interventions were effective in that regard, and the main targeted behaviors to change were lifestyle management, medication intake, self-measurement, and patient-provider interaction.

### Problem Formulation

Despite the availability of effective medications and the prior success of digital interventions, nonadherence to medications still remains a problem for some persons with hypertension. One of the common limitations of digital interventions is that they may be effective in changing the user’s current behavior but cannot adapt to the user’s changing needs over time. Most of the existing digital interventions provide a general solution for their users, and the user himself or herself is responsible for finding the right information or intervention. These digital interventions generally do not consider the barriers causing patients’ nonadherence (and other related behaviors) and their needs over time.

In a previous paper [33], the authors revealed the need to design a multifaceted digital intervention that can be personalized according to one or more patient behaviors that need to be changed to overcome the key determinant or determinants of low adherence to medication or uncontrolled BP among patients with hypertension, considering different levels including patient and health care team and system involvement.

The aim of this study is to design, develop, test, and evaluate a tailored digital intervention, to be delivered through a mobile app, to increase MA and self-care management for persons with hypertension.

## Methods

### Study Design

The design of the study is influenced by the intervention mapping technique [34], which is used for the design and development of health promotion programs. We decided to accomplish such a big study in smaller steps, as follows.

#### Systematic Review

We first conducted a systematic review in order to detect the determinants, behaviors to change, and implemented digital strategies in previous studies [33]. The literature review included key determinants for MA among persons with hypertension. From this review, we have extracted a list of target behaviors and psychological determinants to create an encompassing Matrix of Change Objectives. It reviews digital interventions for persons with hypertension in order to determine which intervention strategies have been employed previously for each combination of behavior and determinant in the Matrix.

#### Focus Groups

We conducted focus groups to ascertain the needs of persons with hypertension in a Swedish context and in relation to the findings of the conducted systematic literature review. The focus groups helped us to determine whether literature findings are applicable to our target population in a Swedish context, whether some key factors must be added or removed from our Matrix of Change, and whether the identified intervention options are applicable and in which order of preference. We will also conduct focus groups after the pilot study to verify the participants’ comprehension of the 16-item Maastrict Utrecht Adherence in Hypertension (MUAH-16) questionnaire regarding MA for hypertension (see Distal Outcomes), translated to Swedish, and also to perform the usability test for the mobile app.

#### App Design

Through what has been learned with the above findings, adaptive digital interventions are designed and developed to be delivered via mobile app.

#### Pilot Study

The pilot intervention will be conducted in order to evaluate the feasibility and usability of the proposed app for 2 weeks with the same group that attended the focus groups.

#### Longitudinal Study

The longitudinal study is designed to assess the effect of the proposed adaptive digital interventions delivered through the mobile app with individuals with hypertension for 6 months. The design is proposed to be an interrupted time series approach, which is considered to be the strongest quasi-experimental design that can be used to evaluate the effectiveness of an intervention [35,36]. The interrupted time series designs start to collect outcome measures before, during, and after intervention steps. Therefore, they are supposed to capture the level and any trend changes of one or more outcomes through time.

We will keep track of multiple variables for each participant through time and try to deliver the right intervention to the right person through patient-reported measures and app usage.

Two arms are considered in this design: One arm receives digital intervention. We will also add a nonequivalent no-treatment arm. This control group will be picked out of the group of patients who fulfilled the inclusion criteria and for whom the only criteria that are available for comparison is the primary MA (ie, pickups from the pharmacy). The reason to add this control group is to be able to address the internal validity threat [37]. By adding the control group, the effect of history is mitigated, and the study is strengthened against other threats to internal validity such as maturation (does the MA improvement occur naturally over time?).

### Recruitment

The study will be performed in Region Halland, Sweden, as it is a collaborative project between Halmstad University and Region Halland. The organization of the health care system in Sweden is designed so that primary care centers are the main actors and there are district nurses to take care of people with high BP registered in each primary care center. There is an integrated electronic health data system available in Region Halland that facilitates the recruitment process.

First, the primary care centers are strategically chosen, and the responsible heads of the primary care centers receive information regarding the project. Through access to the integrated medical data given the ethical approval, we will select the potential pseudonymized persons with hypertension according to the inclusion criteria described below. Then, staff at Region Halland with authority and access to the integrated medical data will reveal their identities. The contact information will be sent to a district nurse working in this study, who will send invitation letters to each eligible person. The letter will contain information about the purpose, that the research objective is to develop an interactive app for persons with hypertension, methodology for the project, instructions explaining how to join, and the informed consent. It will be clearly stated in the invitation letter that participating in the project is voluntary and does not affect the process of treatment in the primary care center.

A week after, the nurse contacts them by telephone regarding participation in the project. The eligible persons are asked to join the project for the first step of the project (focus groups) and will also be invited to join the second step of the project (pilot intervention) and, if wanted, (only) the third step (longitudinal study). Furthermore, being able to speak and understand Swedish and having their own smartphone will also be inclusion criteria that is checked by the nurse.

#### Inclusion Criteria

The inclusion criteria are as follows: (1) aged 40-70 years; (2) have hypertension diagnosis (ie, International Classification of Diseases, Tenth Revision [ICD-10] codes from I10 to I16 in the person’s medical history) for 1 year or more and have prescribed medications; (3) know Swedish, both spoken and written; and (4) have own smartphone.

#### Exclusion Criteria

In order to alleviate the factors that might affect MA and make it hard to see the effect of the intervention, the following exclusion criteria were applied based on experts’ knowledge: (1) receiving medication with unit-dose packaging (Apodos); (2) previous stroke; (3) myocardial infarction; (4) psychological disorder or cognitive impairment (ie, ICD-10 codes F01 to F99); (5) pregnancy-induced hypertension; (6) insulin treatment; or (7) kidney disease defined as glomerular filtration rate <60 mL/min.

Apodos directly affects MA, especially for the patients with polypharmacy. Psychological disorders or cognitive impairments also affect MA directly due to forgetfulness and other factors related to their disease. We believe this type of patient needs specific types of interventions and should not be included in this study. Patients with psychological disorders (ICD-10 F01-F99) will first be excluded based on their medical health records. Patients with any occurrence of these diagnoses, anywhere in their histories, will be excluded. Additionally, though, there might be some cases of psychological disorders that are not registered in electronic health records as diagnoses. Hence, after the interested participants show up, we will also contact the district nurse at the included primary care centers to detect and exclude if such cases exist in the interested participants. Pregnancy-induced hypertension is partially excluded already, since we included only patients who already have at least 1 year of hypertension. The other exclusion criteria (ie, previous stroke, myocardial infarction, insulin treatment, and kidney disorders) were suggested by clinicians to exclude due to having other complications and treatment plans that might interfere with MA. Excluding all, we have around 12,000 individuals with hypertension in Region Halland who may be interested in participating.

### Data Collection and Analysis

#### Focus Groups

In the focus groups (both the ones conducted for needs assessment and the ones for testing the usability and the translated MUAH-16 questionnaire), approximately 6-8 persons with hypertension per group are included in a total of 4-5 groups. The focus groups last approximately 2 hours. Focus groups are chosen to generate a deeper meaning with varying views through group discussions [38]. Due to the outbreak of COVID-19, the possibility of conducting online or telephone focus groups with 2-4 participants is also considered to avoid any possible infection threat in face-to-face focus groups. In order to measure and describe the participants’ acceptability and usability testing and app usage, a semistructured interview guide will be developed based on a mobile health app usability questionnaire [39] (with 3 subscales: ease of use, interface and satisfaction, usefulness). The data from the focus groups will be analyzed with qualitative content analysis and a manifest approach [40].

#### Longitudinal Study

The data from the participants in the study are sent through their mobile app to the server, where all the participants’ data is stored. All the data from the participants are collected via self-reports and their hypertension-relevant information in the aggregated health database. The only item that we measure without the participants’ engagement is if they have seen the intervention and how long it took to answer the questions. This information is used to measure the user’s fatigue and intervention retention and will be used to adapt the interventions.

The effect of the intervention is analyzed statistically using segmented regression analysis, testing for changes in both the level and the trend of the outcome.

### Intervention Design

**Figure 1:**
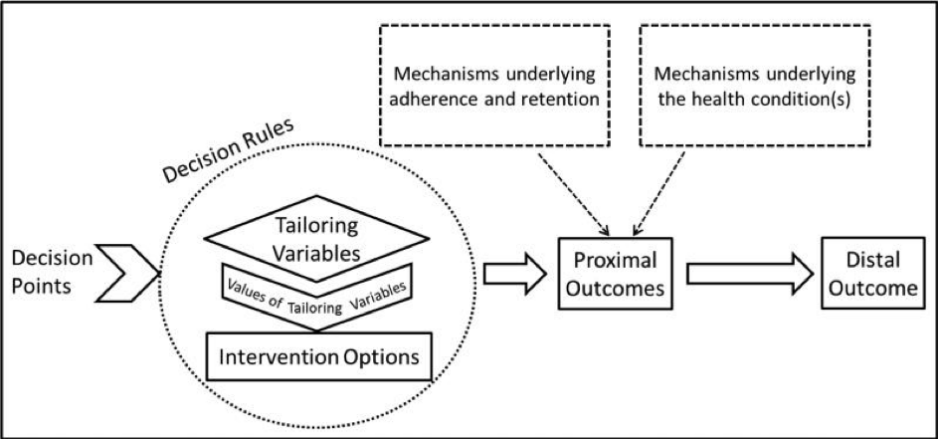
Conceptual model of just-in-time adaptive intervention components.

The design of the digital intervention module is considered to follow the just in-time adaptive intervention framework (JITAI). JITAI is an intervention design aiming to provide the right type and amount of support at the right time by adapting to an individual's context [41]. It has potential for promoting health behavior change, which in our study is supposed to be MA. The conceptual model of JITAI, including all its components, is shown in Figure 1 (borrowed from Nahum-Shani et al [41]). We explain the details of the intervention design following the JITAI conceptual model.

#### Intervention Options

Intervention options are a set of possible interventions that are going to be delivered at decision points. In JITAI, intervention options can be various types of support (information, advice, feedback, etc), source of support (mobile, nurse, physician, etc), amounts of support (intensity, dose, etc), and type of media or channel (phone call, SMS text messaging, etc).

From the conducted literature review, we summarized all types of digital interventions delivered to individuals with hypertension as follows: (1) reminders for medication intake (daily or several times per day depending on the antihypertensive medication plan), reminders for BP measurement (every 3 months), and reminders for physical activity (biweekly); (2) informational contents regarding hypertension and all its facts, consequences, treatments, risks, medications and side effects, lifestyle, and so on (text, videos, etc)—the prepared interventions will be based on health care professional advice plus the reference of webpages for further readings; (3) trends on medication intake, physical activity, etc in a feedback motivational message—based on the recent activity of the user, a feedback motivational message is sent biweekly; and (4) motivational messages—the relevant messages are phrased in a motivated manner (eg, regarding smoking, a message like “Treatment of high blood pressure has a much better effect if you do not smoke.”).

#### Distal Outcomes

Distal outcome is the ultimate goal that the intervention is trying to achieve. In iMedA, we considered primary and secondary distal outcomes. Primarily, we aim at improving MA. Then, in the long term, BP is considered as a secondary distal outcome along with increased quality of life [42], assessment of the lifestyle behavior (smoking, alcohol consumption, physical activity, and food intake) [10], and communicative and critical health literacy [43].

In order to measure MA, we consider the following:

1. Self-reported medication intake through the mobile app is supposed to be collected every day.
2. MUAH-16 [44] is measured before and after the intervention. It is a MA questionnaire for hypertension. It consists of 16 items with four factors: (1) positive attitude towards health care and medication, (2) lack of discipline, (3) aversion toward medication, and (4) active coping with health problems. The items are on a 7-point Likert scale (1=completely disagree to 7=completely agree). A previous study [44] had found correlations between adherence and the MUAH-16 score. Specifically, they found that higher scores in subscale 1 correlated positively with adherence, and higher scores on subscale 2 correlated negatively with adherence.
3. Pickups from pharmacies will be collected after the intervention is finished.

BP is to be measured once before the start of the intervention, at 3 months, and then at 6 months by the recruited nurse.

Health literacy will be measured by the Swedish Communicative and Critical Health Literacy scale. It consists of 5 items with a 5-point Likert scale [43]. Health status will be measured by EQ-5D (EuroQol 5-Dimension questionnaire). It consists of 5 areas for covering health, with 5 response alternatives and 1 overall question regarding health (EQ-VAS [EuroQol visual analogue scale] with score range of 0 to 100) [42]. Both will be measured before and after the intervention.

#### Proximal Outcomes

Proximal outcomes are the short-term goals of the interventions. They can be mediators, intermediate measures, or both for the distal outcomes. The medication intake is considered to be the main proximal outcome, which is measured daily. The physical activity rate, which is measured weekly, is another proximal outcome.

Since most of the contents are educational, and they try to increase the hypertension knowledge of the participants, we will add a proximal outcome to measure how much their knowledge has increased. We consider two methods to measure it. First, after showing the content, we will ask “Did you know...?”. Second, we will design simple gamification tests to be presented to the participants biweekly.

To prevent poor adherence to the interventions, it is recommended to define a few proximal outcomes related to intervention engagement and fatigue. Therefore, we consider the number of clicked interventions, dwell time, number of watched videos, and like/dislike feedbacks for each intervention as proximal outcomes related to intervention adherence and retention.

#### Tailoring Variables

Tailoring variables are information about the participant that is used to decide when to provide which intervention. In other words, they are used to personalize the interventions and make them adaptive to the individual’s circumstances. All proximal outcomes can serve as tailoring variables. They can be measured actively, passively, or both. Active assessments require an individual's engagement in measuring, for example through self-reports, while passive assessments require minimal or no individual engagement, for example through a mobile phone’s sensors.

From baseline information, we can select a few tailoring variables, including alcohol consumption, smoking, specific diet, age, and gender, in order to personalize the interventions. These are considered to be hard-pruning because, with the help of these baseline tailoring variables, a set of intervention options are ruled out from the beginning. As an example, if the person is a nonsmoker, there is no need to motivate him or her to quit smoking.

From the MUAH-16 questionnaire, we start to learn more about an individual’s beliefs, barriers, and behavior about MA. It has 4 subscales regarding (1) positive attitude toward health care and medication, (2) lack of discipline, (3) aversion toward medication, and (4) active coping with health problems. Each subscale contains 4 questions. At the beginning of the intervention, MUAH-16 questions are used as tailoring variables. Then, during the intervention period, and based on the previously delivered informational contents to the individual, the answers to “Did you know...?” questions will be used as tailoring variables.

#### Decision Rules

Decision rules are the adaptation engine of JITAIs. They are used to determine which intervention option to deliver to whom and when. They are the links between intervention options and tailoring variables. Operationally, a JITAI includes a sequence of decision rules (ie, treatment policies) that take the individual’s current context as input and specify whether an intervention should be delivered now and what intervention should be delivered [45].

Decision rules in iMedA will be probabilistic rules from experts modified by “suggestions” from reinforcement learning [46]. Reinforcement learning is used to continuously learn and optimize the treatment policy in JITAI as the individual experiences the interventions. It automatically “discovers” which interventions are most successful for which patients by using statistical machine learning methods.

**Figure 2:**
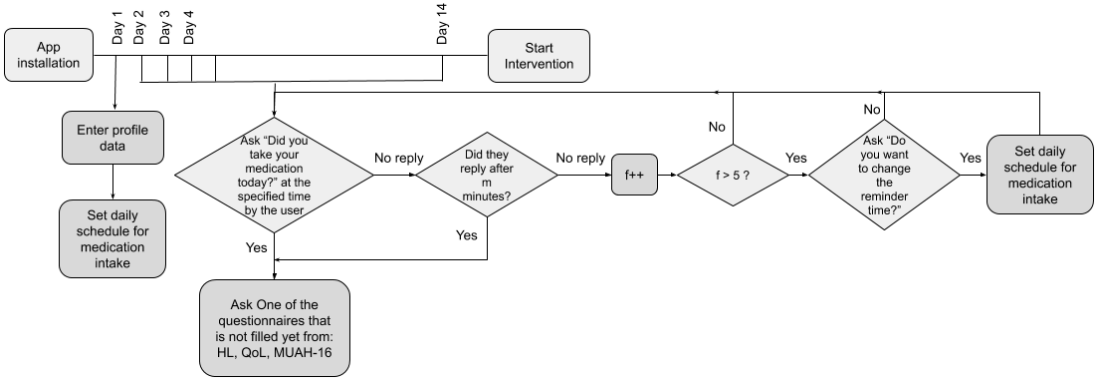
An illustration of the preintervention step (ie, the first 14 days). HL: health literacy; MUAH-16: 16-item Maastricht Utrecht Adherence in Hypertension; QoL: quality of life.

Since we are using an interrupted time series design, we will consider the first 14 days after the app installation to be the preintervention phase (ie, data collection). Figure 2 shows the flowchart of these initial 14 days in more detail.

**Figure 3:**
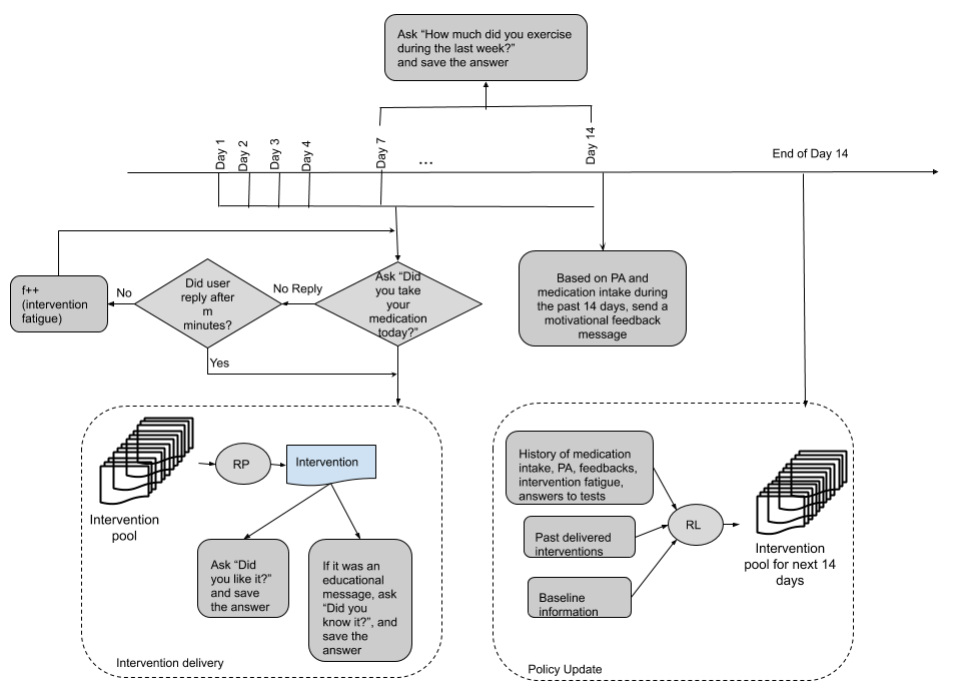
An illustration of the intervention phase. PA: physical activity; RL: reinforcement learning; RP: random policy.

At the beginning of the intervention delivery, the JITAIs will be delivered based on expert suggestions, taking into account the answers to the initial questionnaires, to provide baseline data that a reinforcement learning agent can use to improve upon without the necessity for a warm-up period characterized by random interventions. To allow the agent to learn effectively from collected data, the expert decision rules will be defined in a probabilistic manner, allowing off-policy evaluation. The agent will be built upon a contextual bandit framework that uses tailoring variables to adaptively suggest interventions to improve proximal outcomes and, ultimately, distal outcomes. The agent will optimize the decision rules on a biweekly basis, allowing evaluation before deployment, as illustrated in Figure 3.

##### Contextual Bandit Formulation

The decision rules update is scheduled every 14 days, and the policy (decision rules) remains unchanged during the 2 weeks between each update. With the previous data (tailoring variables, interventions, query-responses) collected, the agent’s policy is updated to personalize interventions for individual patients. We formulate the learning of an optimal policy for a given patient as a stochastic contextual bandit problem. Traditionally, the problem is specified by a tuple (S, A, R) where *S* is the context space (tailoring variables), *A* is the finite action-space (number of interventions), and *R* is the rewards (query-responses). Only rewards of the chosen action are known to the agent at a decision point; rewards of other actions are unknown. Through interactions with the patient, at every decision point at time t, the agent has a sequence of tuples *D* = {(*S*_0_, *A*_0_, *R*_0_), (*S*_1_, *A*_1,_ *R*_1_), ... , (*S_t_*_−1_, *A_t_*_−1_, *R_t_*_−1_)} available to make decisions. *D* is known as the interaction history, containing the context and actions (interventions) chosen by the agent as well as reward received up until *t*−1.

We make several simplifying assumptions necessary for optimal learning:

###### Assumption 1

The stochastic bandit formulation assumes identically and independently distributed contexts, therefore action *A_t_* does not affect the distribution of future contexts *S*_τ_ for τ > *t* + 1.

In simpler terms, the agent does not affect the contexts (tailoring variables) we observe from patients. The effect of each intervention is limited in time, such that we can adjust to changes in behavior gradually.

###### Assumption 2

The expected reward (query-responses) of an action (intervention) can be modeled by an arbitrary (often linear) function *f*:*S* × *A* → *R* for all t such that the expected (average) reward for an action in a particular context can be predicted as:


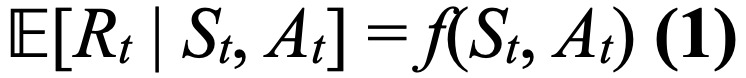


In our formulation, we assume that the context allows an informative mapping from contexts and actions to reward (ie, the reward we would receive for an intervention can be predicted and therefore allows the optimal action to be selected for a particular context).

We cast the learning of function *f*(∙) as a binary classification problem where the probability outputs directly correspond to the expected reward given context *S_t_* and action *A_t_*. At every decision point t, the agent follows a greedy action selection strategy, that is, the agent selects action *A_i_* with the highest predicted reward *f*(*S*_t_, *A_i_*) given the current context:

*A_t_ = π_B_*(*S_t_*) **(2)**,

where *π_B_* is greedy policy, defined as 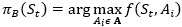.

To facilitate occasional exploration, action *A_t_* is chosen by an *ε*-greedy strategy that randomizes action selection irrespective of current context with probability *ε* ∈ (0,1]. At each time step, either a random action (with probability *ε*) or the action with the maximum predicted reward (with probability 1−*ε*) is chosen.

##### Policy Update

The policy *π_B_* is updated every 14 days using the data collected by the agent thus far by estimating the parameters of function *f*(*S*, *A*). To evaluate new and better policies before deployment, we use importance sampling in combination with the updated reward predictor *f*(*S*, *A*) to estimate the expected reward under the new policies. More formally, given a policy *π_B_* used to collect the past data *D* and the current tuple (*S_t_*, *A_t_*, *R_t_*) forming data set *S* = *D* ∪ (*S_t_*, *A_t_*, *R_t_*), we estimate the expected reward of target policy *π_T_* in combination with the updated reward predictor *f*(*S*, *A*), using the doubly robust estimator [47]:

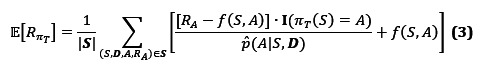



where I(*π_T_*(*S*) = *A*) is an indicator function, being 1 if action *A* is chosen under target policy *π_T_* and 0 otherwise. 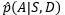 is the probability of choosing an action under the old policy *π_B_*, which is known. For a set of policies Π, we choose the policy that maximizes the average policy reward 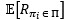:

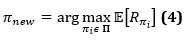



The new policy *π_new_* is then deployed with the *ε*-greedy strategy.

#### Decision Points

A decision point is a time when an intervention decision is made. Considering the nature of JITAIs that are delivered through mobile devices, decision points occur much more rapidly than in standard interventions.

In iMedA, every day the intervention is delivered accompanied by the time of medication intake question (ie, “It is time to take your medication <medication name>. Did you take it today?”). If the patient has >1 antihypertensive dose per day, then the decision point will happen in only one of those reminders.

These daily interventions are chosen from a 14-day intervention list that the reinforcement learning agent has picked. However, the decision rules are updated every 2 weeks, meaning that the reinforcement learning agent will optimize the decision rules to be adapted to the patient's needs after 14 days of looking into the patient's behavior.

#### Sample Size

As a rule of thumb for an interrupted time series, 10 measurement points before and 10 measurements after an intervention provide 80% power to detect a change in level of 5 standard deviations (of the pre-data) only if the autocorrelation (ie, the extent to which data collected close together in time are correlated with each other) is greater than 0.4 [48].

According to simulations done in Liu et al [49], considering the trend change B3=0.1 (B2 + B3 = 0.25), where negative values for the parameters indicate a “decrease” (either level, trend, or both) after intervention, and positive values indicate an “increase” after intervention; autocorrelation >0.4; the Poisson time series; power >0.80; and statistical significance level of 0.05, we need at least 32 participants in the intervention group. Our plan is to recruit 100 participants to the longitudinal study, 50 in each arm due to attrition rate, specifically during the COVID-19 outbreak.

## Results

We have started the focus groups, although the COVID-19 outbreak is a big roadblock at the moment. The design of the digital interventions is in progress, and the mobile app will be ready in November 2020. Then, we are planning to run the pilot study. After fixing all the probable modifications due to the pilot evaluations, the longitudinal study will then start in February 2021. We expect to publish the results of the analysis in mid-2021.

## Discussion

### Overview

This study plans to design, develop, test, and evaluate a mobile app that is personalized and adapted to persons with hypertension-specific needs and behavior in order to increase MA. To do so, it is essential that it is based on theoretical knowledge as well as in contextual settings. Therefore, we start with a literature review to identify these needs and focus groups with persons who will use it.

Conducting the focus groups in different ways, both face-to-face and digital (such as via Skype or telephone) due to the global COVID-19 situation, might create difficulties recruiting participants. Using digital means can also increase bias in the answers and therefore the data. The researchers will make an effort to encourage the participants to describe their experiences. When using digital means, fewer participants can be included each time, which can be positive for some, but it also has a risk of limited discussion [50]. Conversely, having large groups can have a negative impact on some participants’ opportunities to speak [50]. However, using these digital means can also facilitate conducting the focus groups due to the COVID-19 situation.

Since MUAH-16 items have not been translated, tested, and used before in a Swedish context, we aim to perform the process of translation and adaption of this instrument following the World Health Organization guidelines [51]. The forward translation and expert panel back-translation steps have been performed. In order to run the pretesting and cognitive interviewing, to finalize the final version of MUAH-16, the focus groups will be conducted after the pilot study to discuss the items. During the pilot study, the participants are supposed to receive the MUAH-16 items and answer them via the mobile app.

Using focus groups both before and after, including persons with hypertension, will be a strength in the development of the content of the app. It has been stated in other focus groups with persons with hypertension that reminders in an app for MA could be of a positive nature, but on the other hand, their use might cause anxiety [52]. This emphasizes the need to involve persons in the target group in order to develop a personalized app to strengthen self-care management.

### Importance

The intended result of this study is to increase the knowledge of how an interactive app can support MA in persons with hypertension. With the increased limit of health care resources, it is important to use the rapidly growing digital technology to develop new ways of supporting this population as a complement to conventional care. Therefore, to design, develop, test, and evaluate an interactive app used for 6 months with different features is of interest to support persons with hypertension. With different features in an app that is formed as a personalized means of support, it can increase the users’ MA as well as their likelihood to perform lifestyle changes. It is essential to develop features that are appropriate and feasible for the target population in order to conduct a larger study for evaluating the effect.

### Ethical Considerations

This study has been split into smaller studies, for which ethical approval was given by the Swedish Ethical Review Authority (Etikprövningsmyndigheten) (2020/04399, 2019/04067, and 2018/294).

## Abbreviations

BP: blood pressure
EQ-5D: EuroQol 5-Dimension questionnaire
EQ-VAS: EuroQol visual analogue scale
ICD-10: International Classification of Diseases, Tenth Revision
JITAI: just-in-time adaptive intervention
MA: medication adherence
MUAH-16: 16-item Maastrict Utrecht Adherence in Hypertension
